# DYRK1A inhibition suppresses STAT3/EGFR/Met signalling and sensitizes EGFR wild‐type NSCLC cells to AZD9291

**DOI:** 10.1111/jcmm.14609

**Published:** 2019-08-27

**Authors:** Yang‐ling Li, Ke Ding, Xiu Hu, Lin‐wen Wu, Dong‐mei Zhou, Ming‐jun Rao, Neng‐ming Lin, Chong Zhang

**Affiliations:** ^1^ Department of Clinical Pharmacology, Affiliated Hangzhou First People's Hospital Zhejiang University School of Medicine Hangzhou, Zhejiang China; ^2^ School of Medicine Zhejiang University City College Hangzhou Zhejiang China; ^3^ College of Pharmaceutical Sciences Zhejiang University Hangzhou, Zhejiang China; ^4^ Hangzhou Translational Medicine Research Center, Affiliated Hangzhou First People's Hospital Zhejiang University School of Medicine Hangzhou, Zhejiang China

**Keywords:** dual‐specificity tyrosine phosphorylation kinase 1a, epidermal growth factor receptor, Met, non–small‐cell lung cancer, osimertinib

## Abstract

DYRK1A is considered a potential cancer therapeutic target, but the role of DYRK1A in NSCLC oncogenesis and treatment requires further investigation. In our study, high DYRK1A expression was observed in tumour samples from patients with lung cancer compared with normal lung tissues, and the high levels of DYRK1A were related to a reduced survival time in patients with lung cancer. Meanwhile, the DYRK1A inhibitor harmine could suppress the proliferation of NSCLC cells compared to that of the control. As DYRK1A suppression might be effective in treating NSCLC, we next explored the possible specific molecular mechanisms that were involved. We showed that DYRK1A suppression by siRNA could suppress the levels of EGFR and Met in NSCLC cells. Furthermore, DYRK1A siRNA could inhibit the expression and nuclear translocation of STAT3. Meanwhile, harmine could also regulate the STAT3/EGFR/Met signalling pathway in human NSCLC cells. AZD9291 is effective to treat NSCLC patients with EGFR‐sensitivity mutation and T790 M resistance mutation, but the clinical efficacy in patients with wild‐type EGFR remains modest. We showed that DYRK1A repression could enhance the anti‐cancer effect of AZD9291 by inducing apoptosis and suppressing cell proliferation in EGFR wild‐type NSCLC cells. In addition, harmine could enhance the anti‐NSCLC activity of AZD9291 by modulating STAT3 pathway. Finally, harmine could enhance the anti‐cancer activity of AZD9291 in primary NSCLC cells. Collectively, targeting DYRK1A might be an attractive target for AZD9291 sensitization in EGFR wild‐type NSCLC patients.

## 1 INTRODUCTION

Lung cancer is the leading cause of death from cancer worldwide, and the majority of lung cancers (approximately 80%–85%) are non–small‐cell lung cancer (NSCLC).^1^ Much progress has been made recently in personalized therapy for patients with NSCLC.^2^ First‐generation EGF receptor tyrosine kinase inhibitors (EGFR‐TKIs) are most effective in advanced NSCLC patients whose tumours harbour recurrent somatic activating mutations occurring in exons 19 and 21, encoding epidermal growth factor receptor (EGFR), and NSCLC patients with wild‐type EGFR are resistant to EGFR‐TKIs.^3^, ^4^ EGFR T790 M resistance mutation (EGFR T790M) ultimately emerged in most of these patients.^5^ Several third‐generation of EGFR‐TKIs, such as osimertinib (AZD9291), are used for patients with NSCLC who have disease progression after EGFR‐TKI treatment by selectively targeting the T790M mutation. AZD9291 has significantly greater efficacy than that of platinum therapy plus pemetrexed or first‐line EGFR‐TKIs in patients with T790M‐positive advanced NSCLC.^6^, ^7^ However, NSCLC becomes resistance to AZD9291 treatment, and the resistance mechanisms can be divided into EGFR‐independent resistance mechanisms, such as the activation of HER2 or Met, and EGFR‐dependent resistance mechanisms, such as the EGFR C797S mutation.^8^, ^9^

The dual‐specificity tyrosine phosphorylation kinase 1a (DYRK1A) is abnormally expressed in both Down syndrome (DS) and Alzheimer's disease (AD).^10^ The discovery of DYRK1A inhibitors could lead to the invention of a novel therapeutic strategy for DYRK1A‐related diseases such as DS and AD.^11^, ^12^ DYRK1A is also considered a potential anti‐cancer target because it can regulate the cell cycle by affecting both tumour suppressors and oncogenes.^13^ DYRK1A can phosphorylate a plethora of protein targets at their serine or threonine residues, reflecting its role in multiple biological functions.^14^ For example, DYRK1A reduces the level of Cyclin D1 by phosphorylating on Thr286, inducing the proteasomal degradation of Cyclin D1 and cell cycle G₁ phase arrest. Furthermore, DYRK1A suppression can promote the degradation of EGFR and reduce the self‐renewal capacity of glioblastoma cells.^15^ However, whether DYRK1A plays an important role in NSCLC oncogenesis and treatment requires further investigation. In our study, we showed that DYRK1A could positively regulate the STAT3/EGFR/Met signalling pathway in human EGFR wild‐type NSCLC cells, characterized as EGFR‐TKIs‐resistant cells. In addition, DYRK1A suppression by siRNA or an inhibitor could increase the anti‐cancer activity of AZD9291 in EGFR wild‐type NSCLC cells. Our data indicated that targeting DYRK1A might be an attractive target for AZD9291 sensitization in EGFR wild‐type NSCLC patients.

## 2 MATERIALS AND METHODS

### 2.1 Materials

Harmine (cat. no. HY‐N0737A) was obtained from MedChemExpress (Monmouth Junction). AZD9291 (cat. no. S7297) was purchased from Selleck Chemicals.

### 2.2 Cell culture

Human wild‐type EGFR NSCLC cell lines (NCI‐H1299, cat. no. TCHu160; A549, cat. no. TCHu150; NCI‐H460, cat. no. TCHu205) were obtained from Shanghai Institute of Biochemistry and Cell Biology. NCI‐H1299 and NCI‐H460 cells were grown in RPMI‐1640 medium plus 10% foetal bovine serum (FBS), and A549 cells were grown in Ham's F12 medium plus 10% FBS.

### 2.3 Isolation of lung cancer cells from NSCLC patients

Anonymized tumour tissues from patients with NSCLC who underwent surgery were collected with their informed consent, according to the procedures approved by the Ethics Committee at Hangzhou First People's Hospital (REC reference no. 2016/21‐01). Collected NSCLC tumour tissue was placed in cold Ham's F12 medium and transported to the laboratory on ice. Tumour tissue was washed with PBS and minced into 1‐2 mm pieces. Then, the primary NSCLC cancer cells were cultured in Ham's F12 medium plus 15% FBS.

### 2.4 Sulphorhodamine B (SRB) assay

The SRB assay was used to quantify the cell density by measuring the cellular protein content of living cells. First, cancer cells were fixed using 10% TCA solution for 1 hours at 4°C. Next, the cells were rinsed with tap water, dried and stained with 0.4% SRB solution for 30 minutes. Then, the wells were washed with 1% acetic acid five times. Following this, the wells were dried and SRB dye was solubilized with an unbuffered Tris‐based solution. Finally, the optical density was detected at 515 nm using a multiscan spectrum plate reader.

### 2.5 Colony formation assay

Cancer cells (2000 per well) were plated into 6‐well plates and treated with compounds for 14 days to allow colony formation. The medium was replaced with fresh medium containing 10% FBS and compounds every 3 days. After treatment, the medium was removed and cells were washed with PBS 3 times, stained with 1% crystal violet (AMRESCO), washed five times with tap water, allowed to air dry and then were photographed and counted.

### 2.6 Apoptosis detection by propidium iodide (PI) staining

The apoptosis of NSCLC cells was quantified with PI staining as described previously.^16^

### 2.7 SiRNA transfection

DYRK1A siRNA was obtained from GenePharma, and the sense sequences were as follows: DYRK1A‐1 siRNA: 5'‐AUGGAGCUAUGGACGUUAATT‐3'; DYRK1A‐2 siRNA: 5'‐AAACUCGAAUUCAACCUUATT‐3' and negative control siRNA: 5'‐UUCUCCGAACGUGUCACGUTT‐3'. Cells (1 × 10^5^ per well) were plated in 6‐well plates, and the next day, the cells were transfected with DYRK1A siRNA or with negative control siRNA using Lipofectamine 3000 (Invitrogen Corporation) according to the manufacturer's recommendations.

### 2.8 Western blotting

Cell extracts were prepared, and Western blotting analysis was conducted as previously described.^17^ Nuclear extracts were prepared using a Nuclear Protein Extraction Kit (cat. no. R0050, Solarbio). The antibodies used for Western blotting were obtained from different resources: the anti‐Met antibody (cat. no. 8198), anti‐DYRK1A antibody (cat. no. 2771), anti‐phospho‐STAT3 (Tyr705) antibody (cat. no. 9145), anti‐cleaved PARP antibody (cat. no. 9541) and anti‐caspase‐3 antibody (cat. no. 9661) were purchased from Cell Signaling Technology; the anti‐phospho‐AKT (Ser473) antibody (cat. no. sc‐7985), anti‐Mcl‐1 antibody (cat. no. sc‐819), anti‐STAT3 antibody (cat. no. sc‐482), anti‐EGFR antibody (cat. no. sc‐373746) and anti‐GAPDH antibody (cat. no. sc‐25778) were obtained from Santa Cruz Biotechnology; the anti‐Lamin B (cat. no. 12987‐1‐AP) antibody was purchased from Proteintech Group.

### 2.9 Immunofluorescence

NSCLC cells were fixed with 4% paraformaldehyde for 30 minutes, washed with PBS, permeabilized with 0.4% Triton X‐100 and blocked with 2% bovine serum albumin (BSA) in PBS for 30 minutes. An anti‐phospho‐STAT3 (Tyr705) antibody was diluted 1:100 in 0.5% BSA/PBS and applied overnight at 4°C, followed by incubation for 1 hour at room temperature with FITC‐conjugated goat anti‐rabbit IgG (cat. no. 111‐095‐003; Jackson ImmunoResearch) diluted 1:100 in 0.5% BSA/PBS. Cells were then incubated with 0.1% DAPI and examined using a fluorescence microscope.

### 2.10 Statistical analyses

Data points in the figures are shown as the mean ± standard deviation. A two‐tailed Student's *t*‐test is used to determine significant differences. *P < .05; **P < .01; ***P < .001. For the anti‐proliferation assay, combination index (CI) values were calculated by the Calcusyn software, and the mean CI values are presented.^18^ CI < 0.9 indicates synergism; CI 0.9‐1.10 indicates an additive effect; and >1.10 indicates antagonism.

## 3 RESULTS

### 3.1 The overexpression of DYRK1A is related to poor overall survival in lung cancer patients

**Figure 1:**
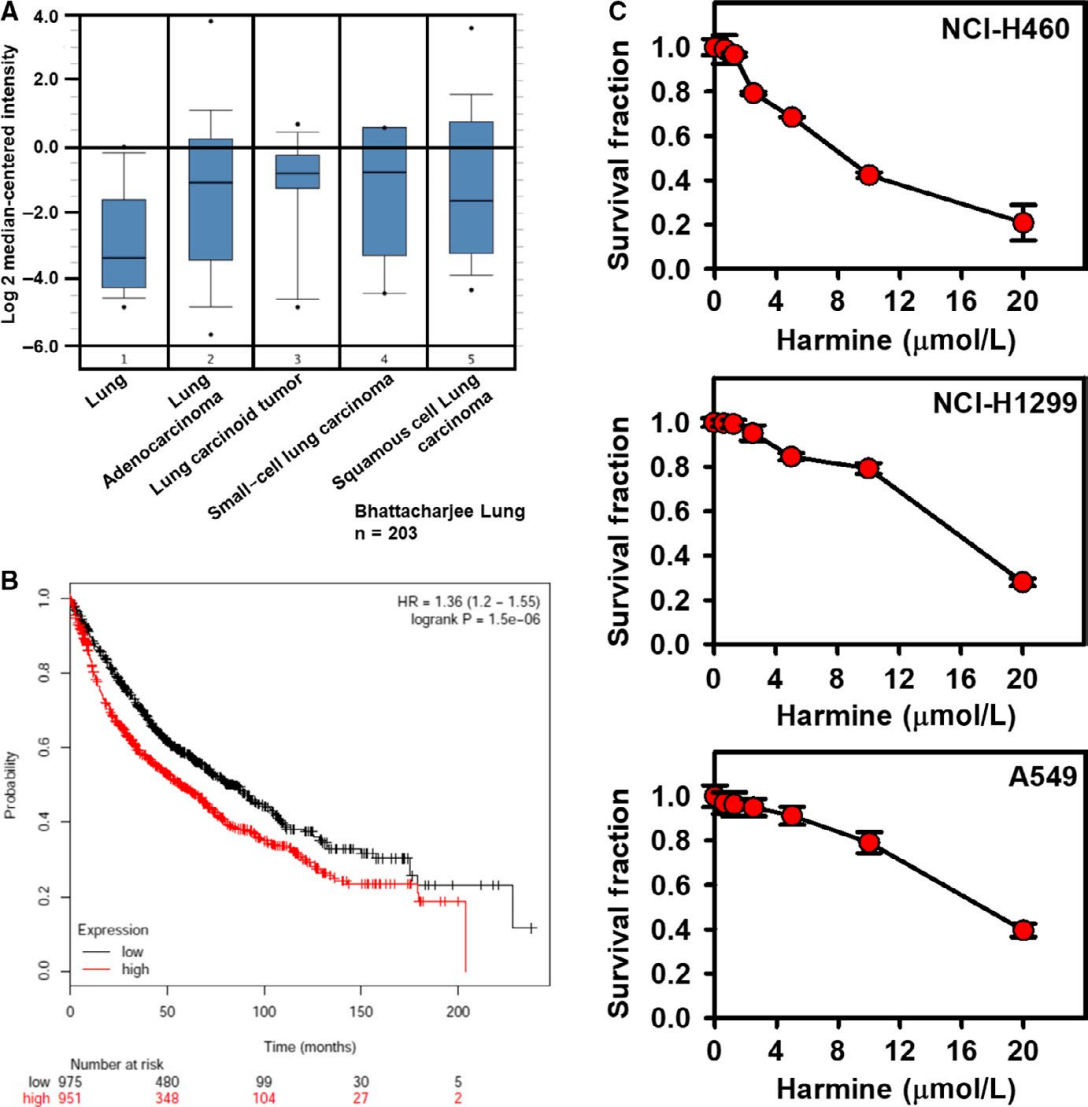
The overexpression of DYRK1A is related to poor overall survival in lung cancer patients, and a DYRK1A inhibitor suppresses the proliferation of NSCLC cells. A, The tumour samples from patients with lung cancer displayed an up‐regulation of DYRK1A compared with that of normal lung tissues (data collected from Oncomine, lung: n = 17; lung adenocarcinoma: n = 132; lung carcinoid tumour: n = 20; small‐cell lung carcinoma: n = 6; squamous cell lung carcinoma: n = 21). Gene: DYRK1A; analysis type: cancer vs normal analysis; data type: mRNA; sample type: clinical specimen; Bhattacharjee Lung; reporter: 36947_s_at.^19^ B, The effects of DYRK1A on the overall survival in patients with NSCLC (data collected from http://kmplot.com/analysis/index.php?p=service&cancer=lung).^20^ C, Lung cancer cells were treated with DYRK1A inhibitor harmine (0.03125, 0.625, 1.25, 2.5, 5, 10 and 20 μmol/L) for 72 h. The SRB assay was used to measure the proliferation of NSCLC cells, including NCI‐H1299, NCI‐H460 and A549 cells

High DYRK1A expression was observed in tumour samples from patients with lung cancer compared with that in normal lung tissues, as shown in Figure 1A (Overexpression gene rank: 177).^19^ Furthermore, we also observed that a high level of DYRK1A was correlated with a reduced survival time in patients with lung cancer using a Kaplan‐Meier plotter analysis (Figure 1B, *P* = 1.5e‐6).^20^ Thus, we hypothesized that DYRK1A might play a critical role in NSCLC progression and that targeting DYRK1A might be an efficient therapeutic strategy to treat patients with NSCLC. In this context, we first demonstrated that the DYRK1A inhibitor harmine could inhibit the proliferation of NSCLC cells in a dose‐dependent manner compared to that of the controls, indicating that targeting DYRK1A might be effective in treating patients with NSCLC (Figure 1C).

### 3.2 DYRK1A regulates STAT3/EGFR/Met signalling in EGFR wild‐type NSCLC cells

**Figure 2:**
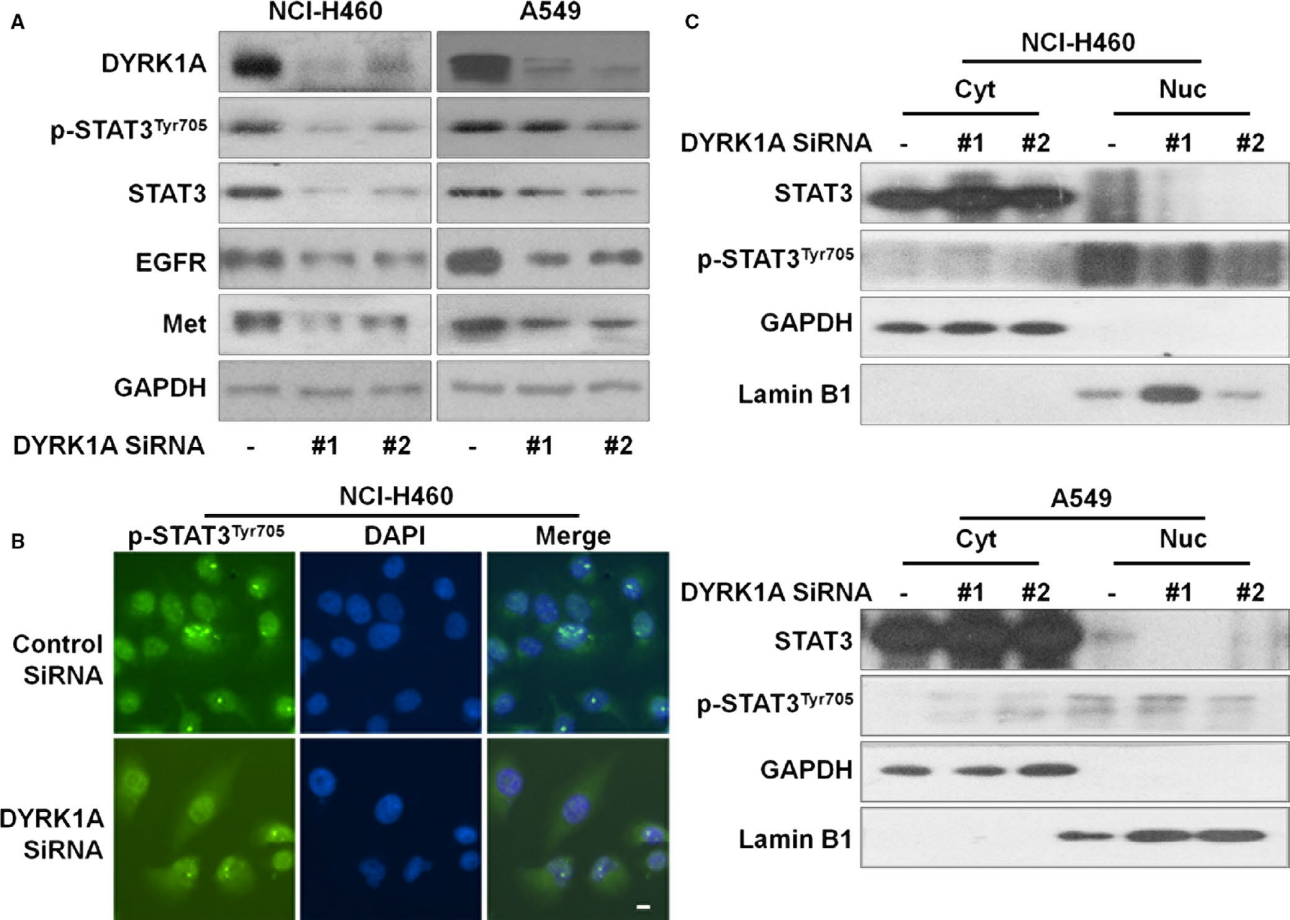
DYRK1A regulates STAT3/EGFR/Met signalling in EGFR wild‐type NSCLC cells. A, DYRK1A knockdown was achieved by transfecting DYRK1A siRNA in NCI‐H460 and A549 cells. Forty‐eight hours after silencing of DYRK1A with siRNA, Western blotting was used to detect the expression of p‐STAT3^Tyr705^, STAT3, EGFR, Met and GAPDH. B, The localization of p‐STAT3^Tyr705^ was determined with immunofluorescence in NCI‐H460 cells. Cells were transfected with DYRK1A siRNA in 96‐well plates. After 48 h, the cells were fixed, permeabilized, and blocked. Finally, the cells were examined using a fluorescence microscope after incubating with p‐STAT3^Tyr705^ antibody and florescence‐labelled secondary antibodies. C, Cells were transfected with DYRK1A siRNA. After 48 h, proteins from the cytosolic and nuclear extracts were separated using Nuclear Protein Extraction Kit, and analysed by Western blotting assay. Cyt, cytosolic extracts; Nuc, nuclear extracts

As DYRK1A suppression might be effective in treating NSCLC, we next explored the possible specific molecular mechanisms involved. First, we found that DYRK1A siRNA could suppress the levels of EGFR and Met receptor tyrosine kinases both in A549 and NCI‐H460 cells (Figure 2A). Thus, we hypothesized that DYRK1A might regulate the STAT3/EGFR/Met pathway in EGFR wild‐type NSCLC cells. Our results indicated that DYRK1A suppression by siRNA could inhibit the expression of STAT3 and indirectly suppress the expression of p‐STAT3^Tyr705^ both in A549 and NCI‐H460 cells (Figure 2A). Furthermore, DYRK1A siRNA blocked the nuclear translocation of p‐STAT3^Tyr705^ in NCI‐H460 cells (Figure 2B). Meanwhile, DYRK1A suppression by siRNA distinctly down‐regulated the levels of STAT3 and indirectly suppressed p‐STAT3^Tyr705^ in the nuclear extractions of NCI‐H460 or A549 cells, indicating that DYRK1A positively regulates STAT3 in NSCLC cells (Figure 2C). These data indicated that DYRK1A could inhibit the STAT3/EGFR/Met pathway.

### 3.3 DYRK1A suppression increases the anti‐cancer activity of AZD9291 in EGFR wild‐type NSCLC cells

**Figure 3:**
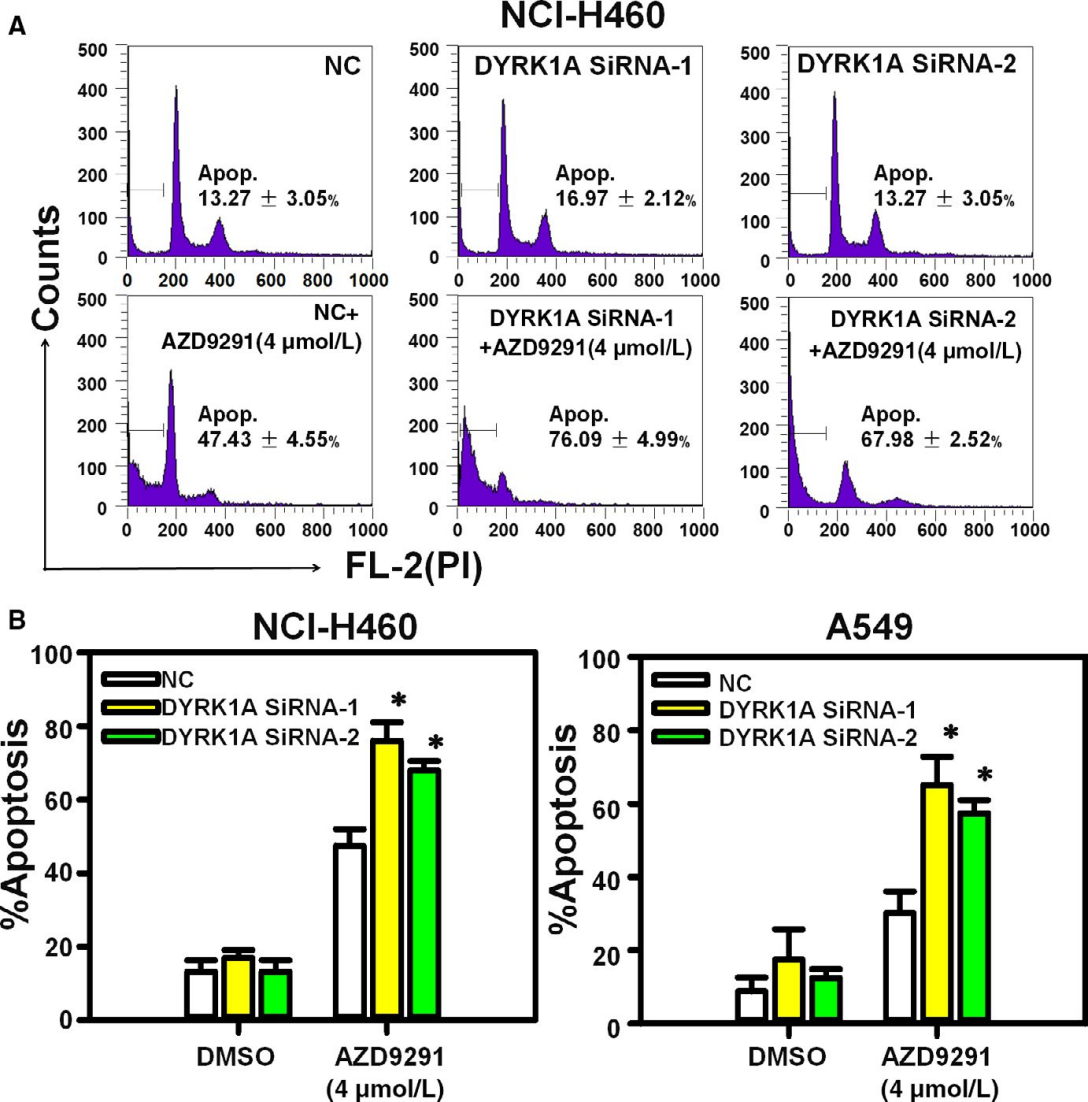
DYRK1A siRNA increases the apoptosis that is induced by AZD9291 in EGFR wild‐type NSCLC cells. A, NCI‐H460 cells were transfected with control siRNA or DYRK1A siRNA and then treated with 4 μmol/L AZD9291 for 48 h. Next, PI staining was used to detect apoptosis. B, NCI‐H460 and A549 cells were transfected with DYRK1A siRNA by and stained with PI, followed by flow cytometry. The experiments were repeated three times, and the error bars represent the standard deviation. DYRK1A siRNA plus AZD9291 vs Control siRNA plus AZD9291, *P < .05

As EGFR‐TKIs are ineffective to treat EGFR wild‐type NSCLC cells, we hypothesized that DYRK1A repression might be an effective strategy for AZD9291 sensitization by suppressing EGFR/Met in EGFR wild‐type NSCLC cells. Figure 3A indicated that DYRK1A siRNA could significantly enhance the apoptosis induced by 4 μmol/L AZD9291 in EGFR wild‐type NSCLC cells. The difference in the number of apoptotic cells between DYRK1A siRNA plus AZD9291 and NC siRNA plus AZD9291 groups reached statistical significance in both NCI‐H460 and A549 cell lines (Figure 3B, *P* < .05).

**Figure 4:**
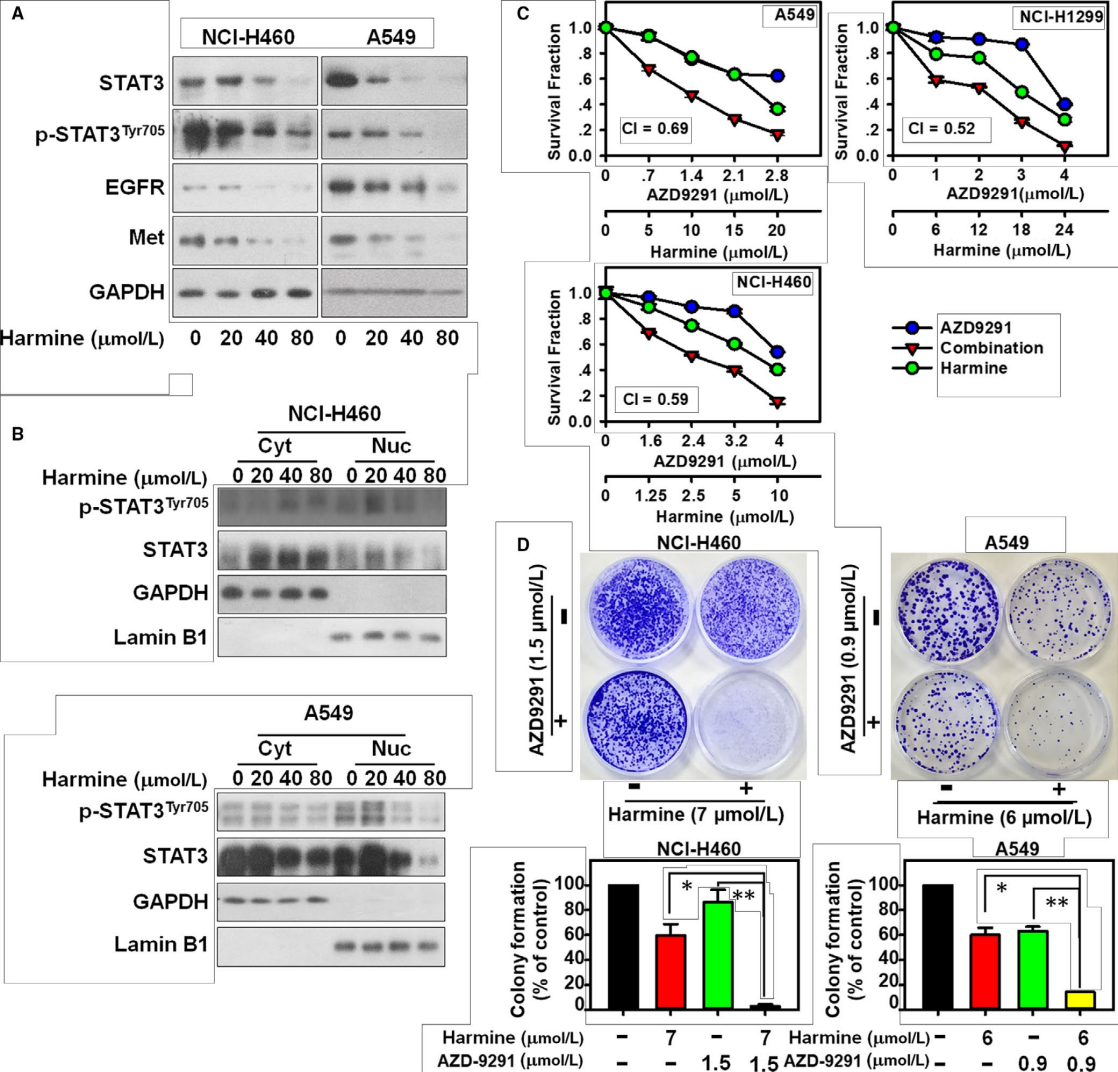
DYRK1A inhibitor harmine sensitizes EGFR wild‐type NSCLC cells to AZD9291 treatment via STAT3/EGFR/Met. A, NCI‐H460 and A549 cells were treated with 0, 20, 40 and 80 μmol/L harmine for 24 h. Western blotting was used to detect the expression of p‐STAT3^Tyr705^, STAT3, EGFR, Met and GAPDH. B, Cells were incubated with 0, 20, 40 and 80 μmol/L harmine for 24 h. The proteins from the cytosolic and nuclear extracts were separated using a Nuclear Protein Extraction Kit, and analysed by Western blotting assay. Cyt, cytosolic extracts; Nuc, nuclear extracts. C, NSCLC cells were treated with harmine and/or AZD9291 at the indicated concentrations for 72 h, and then the proliferation of NSCLC cells was measured with SRB. The mean CI values are shown. D, NCI‐H460 and A549 cells were treated with harmine and/or AZD9291 at the indicated concentrations for 14 days, the cells were then stained with crystal violet and the colony numbers were counted. Harmine plus AZD9291 significantly inhibited NSCLC cell colony formation compared with that of the single agents (*P < .05, **P < .01)

**Figure 5:**
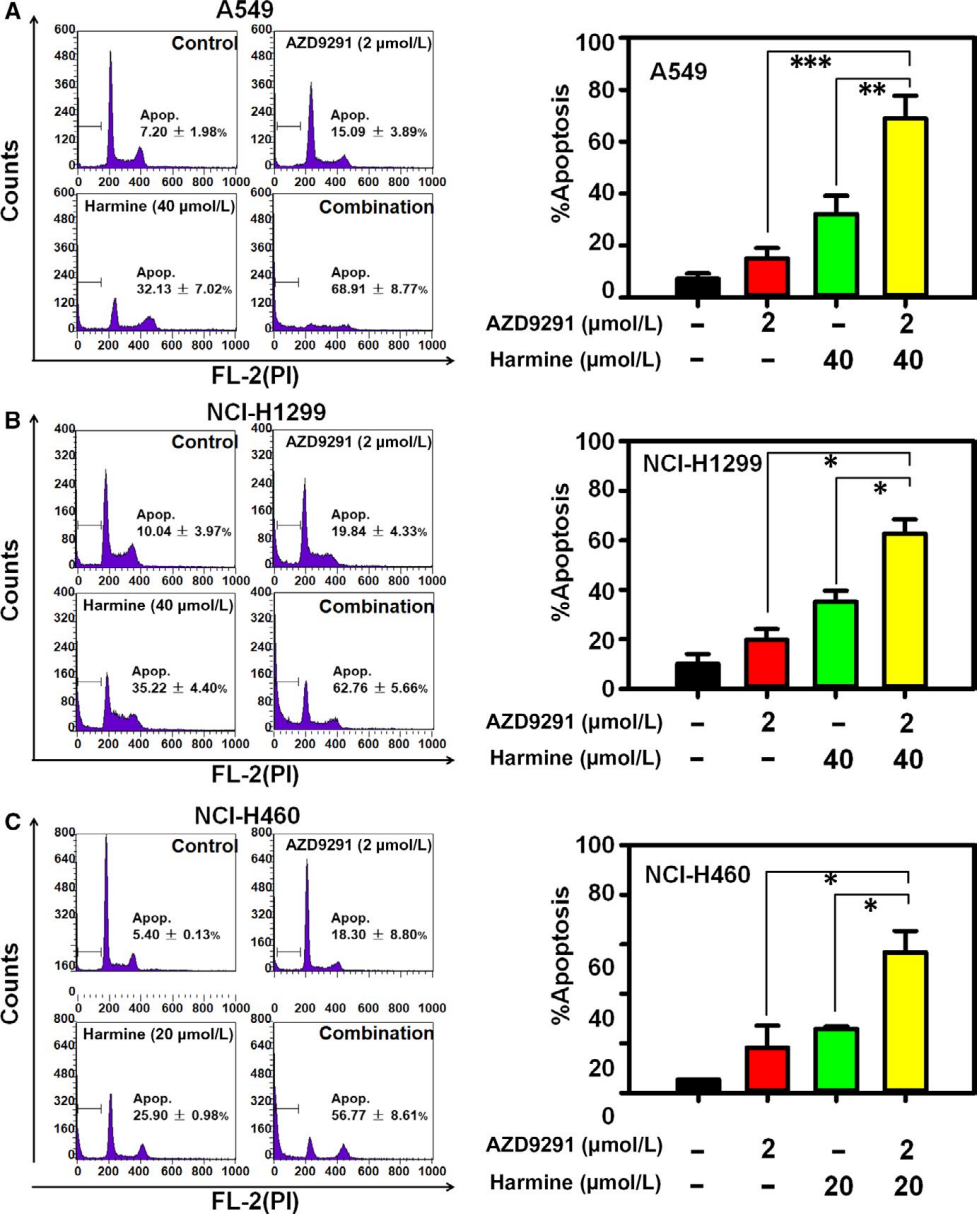
Harmine increases the apoptosis that is induced by AZD9291 in EGFR wild‐type NSCLC cells. A549 (A), NCI‐H1299 (B) and NCI‐H460 (C) cells in six‐well plates were exposed to the compounds for 48 h and then NSCLC cells were analysed by flow cytometry after PI staining. Harmine increased the apoptosis that is induced by AZD9291 in 3 NSCLC cell lines. (*P < .05, **P < .01 and ***P < .001)

Next, our data indicated that the DYRK1A inhibitor harmine could also inhibit the expression of EGFR and Met in a dose‐dependent manner on EGFR wild‐type NSCLC cells (Figure 4A). In addition, harmine significantly inhibited the levels of STAT3 and p‐STAT3^Tyr705^ in the nuclear extractions of NCI‐H460 or A549 cells, indicating that harmine could suppress the activation of STAT3 in EGFR wild‐type NSCLC cells (Figure 4B). Therefore, we hypothesized that harmine might be a sensitizer of AZD9291 in EGFR wild‐type NSCLC treatment by suppressing the STAT3/EGFR/Met pathway. As we expected, harmine could enhance the anti‐proliferation effect of AZD9291 in EGFR wild‐type NSCLC cells, including A549, NCI‐H1299 and NCI‐H460 (Figure 4C). Meanwhile, the analysis of CI value indicated that harmine could synergize with AZD9291 in EGFR wild‐type NSCLC cells (CI < 0.7). In addition, harmine could increase the suppression of the EGFR wild‐type NSCLC cells colony formation that resulted from treatment with AZD9291 in the colony formation assay (Figure 4D). In addition to its synergistic anti‐proliferative effect, the combination of harmine and AZD9291 significantly induced apoptosis in EGFR wild‐type NSCLC cells. In A549 cells, the percentages of the apoptotic cells were vehicle control = 7.20% ± 1.98%, AZD9291 = 15.09% ± 3.89%, harmine = 32.13% ± 7.02% and harmine plus AZD9291 = 68.91% ± 8.77% (harmine plus AZD9291 vs AZD9291, *P* < .001; harmine plus AZD9291 vs harmine, *P* < .01) (Figure 5A). Similarly, combination treatment with harmine and AZD9291 also resulted in increased apoptosis in NCI‐H460 and NCI‐H1299 cells compared to that of monotherapy (Figure 5B and 5). These data indicated that the repression of DYRK1A could increase the anti‐cancer activity of AZD9291 in EGFR wild‐type NSCLC cells.

### 3.4 Harmine sensitizes EGFR wild‐type NSCLC cells to AZD9291 treatment via STAT3

**Figure 6:**
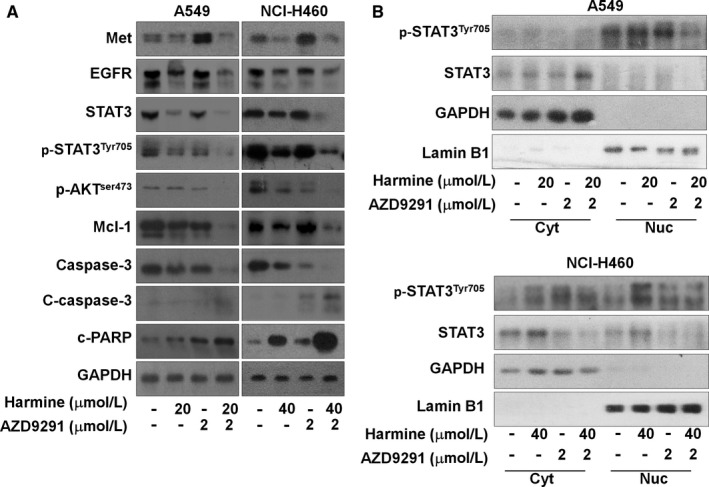
Harmine sensitizes NSCLC cells to AZD9291 treatment via the STAT3/EGFR/Met. A, NCI‐H460 and A549 cells were treated with harmine and/or AZD9291 at the indicated concentrations for 24 h, cells were lysed and proteins were analysed by Western blotting. B, Cells were incubated with harmine and/or AZD9291 at the indicated concentrations for 24 h, proteins from cytosolic and nuclear extracts were separated using Nuclear Protein Extraction Kit, and analysed by Western blotting assay. Cyt, cytosolic extracts; Nuc, nuclear extracts

Our results showed that the activation of caspase‐3 and the cleavage of PARP were clearly observed in the harmine plus AZD9291 group in both A549 and NCI‐H460 cells. Furthermore, Mcl‐1 was significantly suppressed upon treatment with the combination of harmine with AZD9291 but not upon treatment with harmine or AZD9291 alone (Figure 6A). As high Met amplification level is a resistance mechanism to AZD9291, our data demonstrated that AZD9291 increased the expression of Met compared to that of the controls, whereas Met expression was dramatically down‐regulated in the harmine plus AZD9291 combination‐treated NSCLC cells compared to that of monotherapy, indicating that harmine might be an attractive agent to reverse Met overexpression induced by AZD9291. Furthermore, we showed that harmine plus AZD9291 could significantly inhibit STAT3, p‐STAT3^Tyr705^ and the nuclear translocation of STAT3 in NSCLC cells (Figure 6A and B). Collectively, our results suggested that harmine might sensitize the anti‐cancer activity of AZD9291 via the STAT3/Met pathway in EGFR wild‐type NSCLC cells.

### 3.5 Harmine sensitizes primary NSCLC cells to AZD9291 treatment

**Figure 7:**
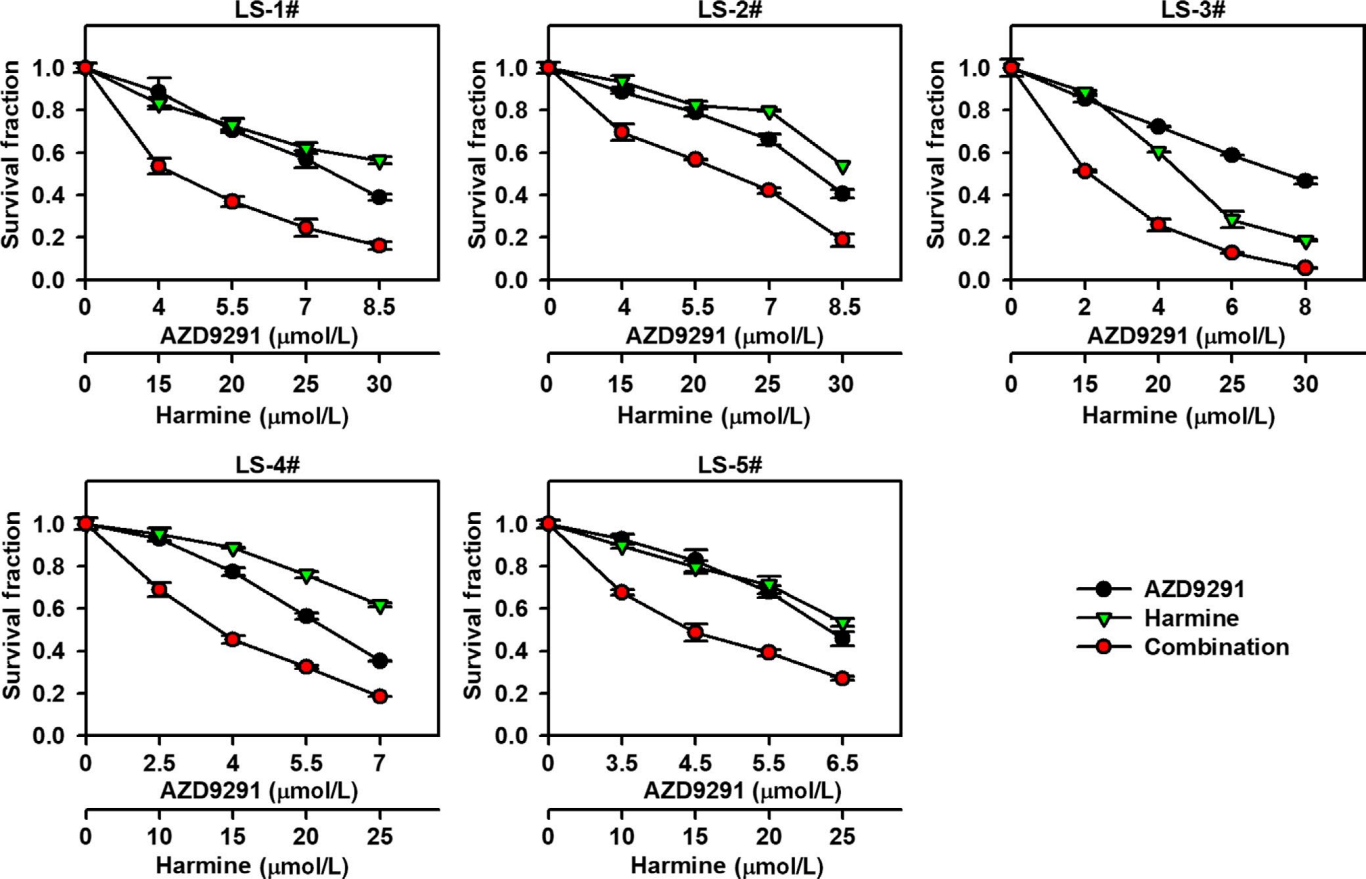
Harmine sensitizes primary NSCLC cells to AZD9291 treatment. Five primary NSCLC cells were incubated with harmine and/or AZD9291 at the indicated concentration for 72 h, and the proliferation of primary NSCLC cells was evaluated by SRB assay

To further validate whether harmine plus AZD9291 could be a therapeutic regimen for patients with NSCLC, we evaluated the anti‐proliferation effects of harmine and/or AZD9291 in five highly aggressive and malignant primary NSCLC cell lines, including two Met‐overexpressing primary NSCLC cell lines (Table S1). As shown in Figure 7, harmine could enhance the anti‐proliferation activity of AZD9291 in highly aggressive and malignant primary NSCLC cells. Here, we provide the first evidence that harmine plus AZD9291 might be an attractive therapeutic strategy for patients with aggressive and highly malignant NSCLC.

## 4 DISCUSSION

DYRK1A is associated with the control of cell growth and tumorigenesis, and it functions as a survival kinase in multiple cancer types, such as cervical cancer, pancreatic cancer and ovarian cancer.^21^ Meanwhile, DYRK1A inhibitors are considered potential candidates for cancer treatment.^14^, ^22^ As the role of DYRK1A played in NSCLC progression has not been identified, the goal of this work was to determine whether DYRK1A is a critical regulator in the development and treatment of NSCLC. Our data showed that high levels of DYRK1A might be related to poor overall survival in patients with lung cancer, and the DYRK1A inhibitor harmine could suppress the proliferation of NSCLC cells. These data indicated that DYRK1A might be an attractive therapeutic target for NSCLC treatment.

The transcription factor STAT3 is overexpressed in multiple cancer types and modulates the expression of genes involved in cell proliferation, survival and differentiation.^23^ Furthermore, STAT3 activation confers cancer cell resistance to anti‐cancer drugs such as trastuzumab and cisplatin, and targeting STAT3 is a potential strategy for sensitizing cancer cells to chemotherapy agents.^24^, ^25^, ^26^, ^27^ STAT3 signalling is also implicated in mediating resistance to EGFR‐TKI therapy, and targeting STAT3 in conjunction with the EGFR results in enhanced antitumour activity.^28^ Thus, we hypothesized that targeting the upstream activators of STAT3 might be an attractive strategy to overcome EGFR‐TKI resistance. DYRK1A overexpression promotes STAT3 activity by phosphorylating STAT3 at Ser727 and contributes to reduced neuronal production and increased astroglial generation in DS.^29^ However, the effect of DYRK1A on STAT3 in cancer cells has not been well demonstrated. In our study, we showed that the down‐regulation of DYRK1A led to the inactivation of STAT3 signalling pathway, indicating that DYRK1A might be a potential upstream activator of STAT3 in NSCLC cells. Furthermore, suppressing DYRK1A with siRNA or with an inhibitor could significantly enhance the anti‐NSCLC activity of AZD9291 via the STAT3/EGFR/Met pathway. The hyperactivation of STAT3 promotes the expression of genes that are involved in cell proliferation (eg Cyclin D1 and c‐Myc), survival (eg Bcl‐XL and Mcl‐1).^23^, ^30^ As DYRK1A could affect the STAT3 pathway in NSCLC, we hypothesized that DYRK1A might also affect the downstream of STAT3. As Met and Mcl‐1 are components of the STAT3 signalling pathway, they are also related to the acquired resistance to AZD9291.^31^, ^32^ Our data showed that DYRK1A suppression could reverse the activation of Met and Mcl‐1 that was induced by AZD9291 in NSCLC cells, indicating that targeting DYRK1A might be effective for overcoming AZD9291 resistance, but further experiments are required to confirm this hypothesis.

EGFR‐TKIs show a clinical benefit for patients with NSCLC harbouring EGFR‐activating mutations; however, NSCLC with wild‐type EGFR unfortunately has a minimal activity to EGFR‐TKIs and chemotherapy remains an important component of treatment.^33^, ^34^ Meanwhile, the majority of patients with NSCLC worldwide are EGFR wild‐type; thus, novel therapeutic strategies are urgently required for treating EGFR wild‐type NSCLC patients.^35^ The combination of EGFR‐TKI with other agent is effective to treat EGFR wild‐type NSCLC patients.^36^, ^37^ In our study, DYRK1A inhibitor harmine plus AZD9291 showed synergistic anti‐cancer activity in EGFR wild‐type NSCLC cell lines, including A549, NCI‐H1299 and NCI‐H460 cells. In addition, this phenomenon is also observed in five primary NSCLC cell lines derived from four EGFR wild‐type NSCLC patients and one patient harbouring EGFR 20ins mutation (Figure 7; Table S1). Those patients with NSCLC can achieve little benefit from EGFR‐TKIs therapy.^38^ Thus, DYRK1A inhibition plus AZD9291 may be effective to treat EGFR wild‐type NSCLC patients. Furthermore, harmine also enhanced the anti‐cancer activity of AZD9291 in HCC827 cells harbouring EGFR Del19 mutation, which are sensitive to AZD9291 treatment (Figure S1). Our study cannot rule out the relationship between the synergistic effect and EGFR mutational status; further clinical investigation is required. Meanwhile, EGFR mutation status may be not a suitable biomarker predicting benefit from such combination regimen.

The amplification and hyperactivation of Met is a resistance mechanism to both first‐ and third‐generation EGFR‐TKIs, and the Met amplification rate is approximately 20% in patients who have acquired resistance to EGFR‐TKIs.^39^, ^40^ As Met is also considered a druggable target in NSCLC, multiple novel EGFR‐Met dual inhibitors have shown potent anti‐cancer activity in acquired resistance NSCLC cells with acquired resistance by targeting both EGFR and Met.^41^, ^42^, ^43^ Furthermore, the combination of an EGFR inhibitor and a Met inhibitor is also considered a logical way to overcome EGFR‐TKI resistance.^44^ In addition, the overexpression of both Met and EGFR is observed in patients with lung cancer, which makes the dual inhibition of Met and EGFR necessary.^45^ In our study, we showed that DYRK1A could positively regulate EGFR and Met in NSCLC cells, and DYRK1A inhibition was efficiently suppressed the transcription of EGFR and Met in A549 cells (Figure S2). Next, we explore how DYRK1A regulates STAT3/EGFR/Met axis. In Figure S3A, STAT3 knockdown inhibited the expression of EGFR and Met in both NCI‐H460 and A549 cells. However, EGFR or Met knockdown could not influence the expression of STAT3, but suppressed the p‐STAT3^Tyr705^ in both NCI‐H460 and A549 (Figure S3B and C). In our study, DYRK1A siRNA could inhibit the expression of STAT3 and indirectly suppress the expression of p‐STAT3^Tyr705^, indicating that DYRK1A suppression may firstly inhibit the expression of STAT3 and then affect the expression of EGFR and Met in EGFR wild‐type NSCLC cells.

Collectively, our study demonstrated that DYRK1A could positively regulate the STAT3/EGFR/Met axis and DYRK1A suppression could enhance the anti‐cancer activity of AZD9291 via STAT3 pathway in EGFR wild‐type NSCLC cells. Meanwhile, DYRK1A inhibitor could also be a chemosensitizing therapeutic agent in combination with AZD9291 treatment.

## CONFLICT OF INTEREST

The authors declare no potential conflicts of interest.

## AUTHORS' CONTRIBUTIONS

YLL, KD, XH, LWW, DMZ and MJR performed the experiments. NML and CZ conceived and designed the study. CZ wrote the manuscript. All authors read and approved the manuscript.

## INFORMED CONSENT

The tumour tissues from patients with NSCLC who underwent surgery were collected with their informed consent, according to the procedures approved by the Ethics Committee at Hangzhou First People's Hospital (REC reference no. 2016/21‐01). The research was carried out according to the World Medical Association Declaration of Helsinki.

## References

[1] **Cho** JH . Immunotherapy for non‐small‐cell lung cancer: current status and future obstacles. Immune Netw. 2017;17:378‐391. 29302251 10.4110/in.2017.17.6.378 PMC5746608

[2] **Hirsch** FR , **Scagliotti** GV , **Mulshine** JL , et al. Lung cancer: current therapies and new targeted treatments. Lancet. 2017;389:299‐311. 27574741 10.1016/S0140-6736(16)30958-8

[3] Cross DA , Ashton SE , Ghiorghiu S , et al. AZD9291, an irreversible EGFR TKI, overcomes T790M‐mediated resistance to EGFR inhibitors in lung cancer. Cancer Discov. 2014;4:1046‐1061. 24893891 10.1158/2159-8290.CD-14-0337 PMC4315625

[4] Li Y‐L , Pan Y‐N , Wu W‐J , et al. Evodiamine induces apoptosis and enhances apoptotic effects of erlotinib in wild‐type EGFR NSCLC cells via S6K1‐mediated Mcl‐1 inhibition. Med Oncol. 2016;33:16. 26757927 10.1007/s12032-015-0726-4

[5] Liao BC , Lin CC , Yang JC . Second and third‐generation epidermal growth factor receptor tyrosine kinase inhibitors in advanced nonsmall cell lung cancer. Curr Opin Oncol. 2015;27:94‐101. 25611025 10.1097/CCO.0000000000000164

[6] Mok TS , Wu YL , Ahn MJ , et al. Osimertinib or platinum‐pemetrexed in EGFR T790M‐positive lung cancer. N Engl J Med. 2017;376:629‐640. 27959700 10.1056/NEJMoa1612674 PMC6762027

[7] Soria JC , Ohe Y , Vansteenkiste J , et al. Osimertinib in untreated EGFR‐mutated advanced non‐small‐cell lung cancer. N Engl J Med. 2018;378:113‐125. 29151359 10.1056/NEJMoa1713137

[8] Thress KS , Paweletz CP , Felip E , et al. Acquired EGFR C797S mutation mediates resistance to AZD9291 in non‐small cell lung cancer harboring EGFR T790M. Nat Med. 2015;21:560‐562. 25939061 10.1038/nm.3854 PMC4771182

[9] Tang ZH , Lu JJ . Osimertinib resistance in non‐small cell lung cancer: mechanisms and therapeutic strategies. Cancer Lett. 2018;420:242‐246. 29425688 10.1016/j.canlet.2018.02.004

[10] Neumann F , Gourdain S , Albac C , et al. DYRK1A inhibition and cognitive rescue in a Down syndrome mouse model are induced by new fluoro‐DANDY derivatives. Sci Rep. 2018;8:2859. 29434250 10.1038/s41598-018-20984-z PMC5809559

[11] Ogawa Y , Nonaka Y , Goto T , et al. Development of a novel selective inhibitor of the Down syndrome‐related kinase Dyrk1A. Nat Commun. 2010;1:86. 20981014 10.1038/ncomms1090

[12] Coutadeur S , Benyamine H , Delalonde L , et al. A novel DYRK1A (dual specificity tyrosine phosphorylation‐regulated kinase 1A) inhibitor for the treatment of Alzheimer's disease: effect on Tau and amyloid pathologies in vitro. J Neurochem. 2015;133:440‐451. 25556849 10.1111/jnc.13018

[13] Jarhad DB , Mashelkar KK , Kim HR , et al. Dual specificity tyrosine‐phosphorylation‐regulated kinase 1a (DYRK1a) kinase inhibitors as potential therapeutics. J Med Chem. 2018;61:9791‐9810. 29985601 10.1021/acs.jmedchem.8b00185

[14] Nguyen TL , Fruit C , Hérault Y , Meijer L , Besson T . Dual‐specificity tyrosine phosphorylation‐regulated kinase 1A (DYRK1A) inhibitors: a survey of recent patent literature. Expert Opin Ther Pat. 2017;27:1183‐1199. 28766366 10.1080/13543776.2017.1360285

[15] Pozo N , Zahonero C , Fernández P , et al. Inhibition of DYRK1A destabilizes EGFR and reduces EGFR‐dependent glioblastoma growth. J Clin Investig. 2013;123:2475‐2487. 23635774 10.1172/JCI63623 PMC3668845

[16] Zhang C , Shi J , Mao S‐Y , et al. Role of p38 MAPK in enhanced human cancer cells killing by the combination of aspirin and ABT‐737. J Cell Mol Med. 2015;19:408‐417. 25388762 10.1111/jcmm.12461 PMC4407609

[17] Hu X , Wu LW , Zhang ZY , et al. The anti‐tumor effect of regorafenib in lung squamous cell carcinoma in vitro. Biochem Biophys Res Comm. 2018;503:1123‐1129. 29944884 10.1016/j.bbrc.2018.06.129

[18] Chou TC , Talalay P . Quantitative analysis of dose‐effect relationships: the combined effects of multiple drugs or enzyme inhibitors. Adv Enzyme Regul. 1984;22:27‐55. 6382953 10.1016/0065-2571(84)90007-4

[19] Bhattacharjee A , Richards WG , Staunton J , et al. Classification of human lung carcinomas by mRNA expression profiling reveals distinct adenocarcinoma subclasses. Proc Natl Acad Sci USA. 2001;98:13790‐13795. 11707567 10.1073/pnas.191502998 PMC61120

[20] Győrffy B , Surowiak P , Budczies J , Lánczky A . Online survival analysis software to assess the prognostic value of biomarkers using transcriptomic data in non‐small‐cell lung cancer. PLoS One. 2013;8:e82241. 24367507 10.1371/journal.pone.0082241 PMC3867325

[21] Guo X , Williams JG , Schug TT , Li X . DYRK1A and DYRK3 promote cell survival through phosphorylation and activation of SIRT1. J Biol Chem. 2010;285:13223‐13232. 20167603 10.1074/jbc.M110.102574 PMC2857074

[22] Fernandez‐Martinez P , Zahonero C , Sanchez‐Gomez P . DYRK1A: the double‐edged kinase as a protagonist in cell growth and tumorigenesis. Mol Cell Oncol. 2015;2:e970048. 27308401 10.4161/23723548.2014.970048 PMC4905233

[23] Laudisi F , Cherubini F , Monteleone G , Stolfi C . STAT3 interactors as potential therapeutic targets for cancer Treatment. Int J Mol Sci. 2018;19:1787. 10.3390/ijms19061787 PMC6032216 29914167

[24] Wang L , Wang Q , Gao M , et al. STAT3 activation confers trastuzumab‐emtansine (T‐DM1) resistance in HER2‐positive breast cancer. Cancer Sci. 2018;109:3305–3315. 30076657 10.1111/cas.13761 PMC6172075

[25] Jin P , Liu Y , Wang R . STAT3 regulated miR‐216a promotes ovarian cancer proliferation and cisplatin resistance. Biosci Rep. 2018;38:BSR20180547. 30061175 10.1042/BSR20180547 PMC6131203

[26] Lankadasari MB , Aparna JS , Mohammed S , et al. Targeting S1PR1/STAT3 loop abrogates desmoplasia and chemosensitizes pancreatic cancer to gemcitabine. Theranostics. 2018;8:3824‐3840. 30083262 10.7150/thno.25308 PMC6071521

[27] Huang LL , Rao W . SiRNA interfering STAT3 enhances DDP sensitivity in cervical cancer cells. Eur Rev Med Pharmacol Sci. 2018;22:4098‐4106. 30024597 10.26355/eurrev_201807_15401

[28] Zulkifli AA , Tan FH , Putoczki TL , Stylli SS , Luwor RB . STAT3 signaling mediates tumour resistance to EGFR targeted therapeutics. Mol Cell Endocrinol. 2017;451:15‐23. 28088467 10.1016/j.mce.2017.01.010

[29] Kurabayashi N , Nguyen MD , Sanada K . DYRK1A overexpression enhances STAT activity and astrogliogenesis in a Down syndrome mouse model. EMBO Rep. 2015;16:1548‐1562. 26373433 10.15252/embr.201540374 PMC4641506

[30] Bhattacharya S , Ray RM , Johnson LR . STAT3‐mediated transcription of Bcl‐2, Mcl‐1 and c‐IAP2 prevents apoptosis in polyamine‐depleted cells. Biochem J. 2005;392:335‐344. 16048438 10.1042/BJ20050465 PMC1316269

[31] Shi P , Oh Y‐T , Deng L , et al. Overcoming acquired resistance to AZD9291, a third‐generation EGFR inhibitor, through modulation of MEK/ERK‐dependent Bim and Mcl‐1 degradation. Clin Cancer Res. 2017;23:6567‐6579. 28765329 10.1158/1078-0432.CCR-17-1574 PMC5668147

[32] Ortiz‐Cuaran S , Scheffler M , Plenker D , et al. Heterogeneous mechanisms of primary and acquired resistance to third‐generation EGFR inhibitors. Clin Cancer Res. 2016;22:4837‐4847. 27252416 10.1158/1078-0432.CCR-15-1915

[33] Wu D‐W , Chen T‐C , Huang H‐S , Lee H . TC‐N19, a novel dual inhibitor of EGFR and cMET, efficiently overcomes EGFR‐TKI resistance in non‐small‐cell lung cancer cells. Cell Death Dis. 2016;7:e2290. 27362807 10.1038/cddis.2016.192 PMC5108342

[34] Heineman DJ , Daniels JM , Schreurs WH . Clinical staging of NSCLC: current evidence and implications for adjuvant chemotherapy. Ther Adv Med Oncol. 2017;9:599‐609. 29081843 10.1177/1758834017722746 PMC5564882

[35] Garon EB , Siegfried JM , Stabile LP , et al. Randomized phase II study of fulvestrant and erlotinib compared with erlotinib alone in patients with advanced or metastatic non‐small cell lung cancer. Lung Cancer. 2018;123:91‐98. 30089602 10.1016/j.lungcan.2018.06.013 PMC6118115

[36] Morgillo F , Fasano M , Della Corte CM , et al. Results of the safety run‐in part of the METAL (METformin in Advanced Lung cancer) study: a multicentre, open‐label phase I‐II study of metformin with erlotinib in second‐line therapy of patients with stage IV non‐small‐cell lung cancer. ESMO Open. 2017;2:e000132. 28761738 10.1136/esmoopen-2016-000132 PMC5519802

[37] Raimbourg J , Joalland M‐P , Cabart M , et al. Sensitization of EGFR wild‐type non‐small cell lung cancer cells to EGFR‐tyrosine kinase inhibitor erlotinib. Mol Cancer Ther. 2017;16:1634‐1644. 28522592 10.1158/1535-7163.MCT-17-0075

[38] Xu J , Jin B , Chu T , et al. EGFR tyrosine kinase inhibitor (TKI) in patients with advanced non‐small cell lung cancer (NSCLC) harboring uncommon EGFR mutations: a real‐world study in China. Lung Cancer. 2016;96:87‐92. 27133756 10.1016/j.lungcan.2016.01.018

[39] Shi P , Oh Y‐T , Zhang G , et al. Met gene amplification and protein hyperactivation is a mechanism of resistance to both first and third generation EGFR inhibitors in lung cancer treatment. Cancer Lett. 2016;380:494‐504. 27450722 10.1016/j.canlet.2016.07.021

[40] Bean J , Brennan C , Shih J‐Y , et al. MET amplification occurs with or without T790M mutations in EGFR mutant lung tumors with acquired resistance to gefitinib or erlotinib. Proc Natl Acad Sci USA. 2007;104:20932‐20937. 18093943 10.1073/pnas.0710370104 PMC2409244

[41] Kim S , Kim TM , Kim D‐W , et al. Acquired resistance of MET‐amplified non‐small cell lung cancer cells to the MET inhibitor capmatinib. Cancer Res Treat. 2018;51:951–962. 30309221 10.4143/crt.2018.052 PMC6639226

[42] Jung SK , Lee M‐H , Lim DY , et al. Butein, a novel dual inhibitor of MET and EGFR, overcomes gefitinib‐resistant lung cancer growth. Mol Carcinog. 2015;54:322‐331. 24974831 10.1002/mc.22191

[43] Singh PK , Silakari O . Novel EGFR (T790M)‐cMET dual inhibitors: putative therapeutic agents for non‐small‐cell lung cancer. Future Med Chem. 2017;9:469‐483. 28362115 10.4155/fmc-2016-0234

[44] Yamaoka T , Ohmori T , Ohba M , et al. Acquired resistance mechanisms to combination Met‐TKI/EGFR‐TKI exposure in met‐amplified EGFR‐TKI‐resistant lung adenocarcinoma harboring an activating EGFR mutation. Mol Cancer Ther. 2016;15:3040‐3054. 27612490 10.1158/1535-7163.MCT-16-0313

[45] Mataki H , Seki N , Chiyomaru T , et al. Tumor‐suppressive microRNA‐206 as a dual inhibitor of MET and EGFR oncogenic signaling in lung squamous cell carcinoma. Int J Oncol. 2015;46:1039‐1050. 25522678 10.3892/ijo.2014.2802

